# The complete chloroplast genome sequence of *Xylia xylocarpa*

**DOI:** 10.1080/23802359.2020.1715297

**Published:** 2020-01-20

**Authors:** Yunfen Geng, Yunqing Li, Jinfeng Zhang, Yi Wang

**Affiliations:** Laboratory of Forest Plant Cultivation and Utilization, Yunnan Academy of Forestry, Kunming, People’s Republic of China

**Keywords:** *Xylia xylocarpa*, chloroplast, Illumina sequencing, phylogenetic analysis

## Abstract

The first complete chloroplast genome (cpDNA) sequence of *Xylia xylocarpa* was determined from Illumina HiSeq pair-end sequencing data in this study. The cpDNA is 161,288 bp in length, contains a large single copy region (LSC) of 89,186 bp and a small single copy region (SSC) of 19,354 bp, which were separated by a pair of inverted repeats (IR) regions of 26,370 bp. The genome contains 131 genes, including 86 protein-coding genes, eight ribosomal RNA genes, and 37 transfer RNA genes. The overall GC content of the whole genome is 36.6%, and the corresponding values of the LSC, SSC, and IR regions are 34.1%, 30.8%, and 42.8%, respectively. Further phylogenomic analysis showed that *X. xylocarpa* clustered in a unique clade in Caesalpinioideae subfamily.

*Xylia xylocarpa* belongs to the genus of *Xylia* within the Fabaceae and distributes naturally in India, Indo-China, Myanmar, and Thailand (Wattanakulpakin et al. 2015). It is a fast-growing tree with a denser wood and yields a hard and durable wood used for heavy construction (Josue 2004). The mature seed can provide a variety of nutrients such as protein, fat, and fiber (Siddhuraju et al. 1995). The extracts of *X. xylocarpa* also showed anti-cholinesterase and memory-improving effects (Lam et al. 2016). However, there have been no genomic studies on *X. xylocarpa*.

Herein, we reported and characterized the complete *X. xylocarpa* plastid genome. The GenBank accession number is MN823700. One *X. xylocarpa* individual (specimen number: 201907030) was collected from Puwen, Yunnan Province of China (23°30′38″N, 101°36′47″E). The specimen is stored at Yunnan Academy of Forestry Herbarium, Kunming, China, and the accession number is ZJFEP119. DNA was extracted from its fresh leaves using DNA Plantzol Reagent (Invitrogen, Carlsbad, CA, USA).

Paired-end reads were sequenced using Illumina HiSeq system (Illumina, San Diego, CA). In total, about 26.8 million high-quality clean reads were generated with adaptors trimmed. Aligning, assembly, and annotation were conducted using CLC de novo assembler (CLC Bio, Aarhus, Denmark), BLAST, GeSeq (Tillich et al. 2017), and GENEIOUS v 11.0.5 (Biomatters Ltd, Auckland, New Zealand). To confirm the phylogenetic position of *X. xylocarpa*, other five species of *Caesalpinioideae* subfamily from NCBI were aligned using MAFFT v.7 (Katoh and Standley 2013). The Auto algorithm in the MAFFT alignment software was used to align the eight complete genome sequences and the G-INS-i algorithm was used to align the partial complex sequences. The maximum-likelihood (ML) bootstrap analysis was conducted using RAxML (Stamatakis 2006); bootstrap probability values were calculated from 1000 replicates. *Stylosanthes hamata* (MG735673) and *Arachis batizocoi* (MK144820) were served as the out-group.

The complete *X. xylocarpa* plastid genome is a circular DNA molecule with the length of 161,288 bp, contains a large single copy region (LSC) of 89,186 bp and a small single copy region (SSC) of 19,354 bp, which were separated by a pair of inverted repeats (IR) regions of 26,370 bp. The overall GC content of the whole genome is 36.6%, and the corresponding values of the LSC, SSC, and IR regions are 34.1%, 30.8%, and 42.8%, respectively. The plastid genome contained 131 genes, including 86 protein-coding genes, eight ribosomal RNA genes, and 37 transfer RNA genes. Phylogenetic analysis showed that *X. xylocarpa* clustered in a unique clade in *Caesalpinioideae* subfamily (Figure 1). The determination of the complete plastid genome sequences provided new molecular data to illuminate the *Caesalpinioideae* subfamily evolution.

**Figure 1.**
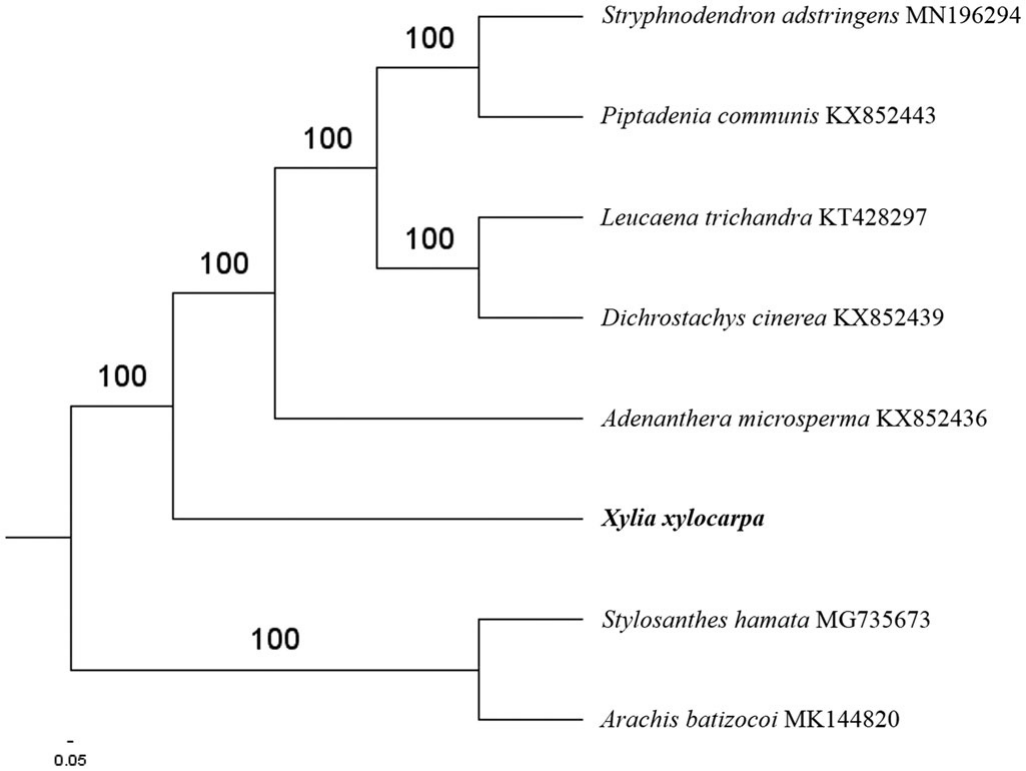
The maximum-likelihood tree based on the six chloroplast genomes of *Caesalpinioideae* subfamily. The bootstrap value based on 1000 replicates is shown on each node.
